# Clinical characteristics and predictors for in-hospital mortality in adult COVID-19 patients: A retrospective single center cohort study in Vilnius, Lithuania

**DOI:** 10.1371/journal.pone.0290656

**Published:** 2023-08-25

**Authors:** Ieva Kubiliute, Monika Vitkauskaite, Jurgita Urboniene, Linas Svetikas, Birute Zablockiene, Ligita Jancoriene

**Affiliations:** 1 Clinic of Infectious Diseases and Dermatovenerology, Institute of Clinical Medicine, Faculty of Medicine, Vilnius University, Vilnius, Lithuania; 2 Faculty of Medicine, Vilnius University, Vilnius, Lithuania; 3 Center of Infectious Diseases, Vilnius University Hospital Santaros Clinics, Vilnius, Lithuania; Dhaka Medical College and Hospital, BANGLADESH

## Abstract

**Background:**

The COVID-19 infection had spread worldwide causing many deaths. Mortality rates and patients’ characteristics varied within and between countries, making it important to understand the peculiarities of different populations. The aim of this study was to identify the main predictors associated with in-hospital mortality due to COVID-19 in Vilnius, Lithuania.

**Materials and methods:**

This was a retrospective observational cohort study conducted at Vilnius University Hospital Santaros Clinics, Lithuania. The study included SARS-CoV-2 positive patients aged over 18 years and hospitalized between March 2020 and May 2021. Depersonalized data were retrieved from electronic medical records. The predictive values of laboratory parameters were evaluated using ROC analysis. Multivariable binary logistic regression was performed to reveal predictors of in-hospital mortality due to COVID-19.

**Results:**

Among 2794 patients, 54.4% were male, the age median was 59 years (IQR 48–70), 47.4% had at least one comorbidity. The most common comorbidities were arterial hypertension (36.9%) and diabetes mellitus (13.7%). Overall, 12.7% of patients died. Multivariable regression revealed that age (OR 1.04, 95%CI 1.02–1.06), congestive heart failure (OR 3.06, 95%CI 1.96–4.77), obesity (OR 3.90, 95%CI 2.12–7.16), COPD (OR 2.92, 95%CI 1.12–7.60), previous stroke (OR 5.80, 95%CI 2.07–16.21), urea >7.01 mmol/l (OR 2.32, 95%CI 1.47–3.67), AST/ALT >1.49 (OR 1.54, 95%CI 1.08–2.21), LDH >452.5 U/l (OR 2.60, 95%CI 1.74–3.88), CRP >92.68 mg/l (OR 1.58, 95%CI 1.06–2.35), IL-6 >69.55 ng/l (OR 1.62, 95%CI 1.10–2.40), and troponin I >18.95 ng/l (OR 2.04, 95%CI 1.38–3.02), were associated with increased risk for in-hospital mortality in COVID-19 patients.

**Conclusions:**

Age, congestive heart failure, obesity, COPD, prior stroke, and increased concentration of urea, LDH, CRP, IL-6, troponin I, ALT to AST ratio were identified to be the predictors for in-hospital mortality of COVID-19 patients.

## Introduction

In December 2019, the first atypical pneumonia cases of unknown etiology were identified in Wuhan City, China [1, 2]. The virus causing this disease was subsequently confirmed as belonging to the β group of coronaviruses and was described as Severe Acute Respiratory Syndrome Corona Virus 2 (SARS-CoV-2) by the International Committee on Taxonomy of Viruses (ICTV) [3, 4]. Due to its rapid global spread, the World Health Organization (WHO) declared the outbreak of the novel coronavirus a pandemic on 11 March 2020 [5]. Over 169 million cases of COVID-19 infection have been confirmed worldwide by 1 June 2021, including more than 3.53 million deaths [6].

SARS-CoV-2 is mainly transmitted between people through respiratory droplets and aerosols [7]. The virus primarily targets tissues of both the upper and lower airway by utilizing angiotensin-converting enzyme (ACE2) and the transmembrane protease serine 2 (TMPRSS2) which are co-expressed in most epithelial cells of the human respiratory tract. COVID-19 has a broad spectrum of clinical manifestation, ranging from asymptomatic to severe disease with multiple organ failure [7, 8]. Hospitalization rates vary between 4 and 7% in different population settings [7]. Moreover, studies have found that up to 44% of hospitalized patients were admitted to the intense care unit and at least one third of them died between March 2020 and January 2021 [9–12]. Patient characteristics and mortality rates vary substantially within and between countries and over time due to a lack of standardized testing strategies, different hospital admission criteria and unequal capacities of health care systems [13, 14]. The geriatric population, people with major comorbidities, and males were considered to be at higher risk of poor clinical outcome, longer hospital stays and higher mortality rate [15–17]. Patients with hypertension, diabetes mellitus, obesity and chronic kidney disease have been observed at the high risk for mortality in COVID-19 [13, 18]. These comorbidities are not only associated with a downregulation of immune system but also with a substantially increased expression of ACE2. The latter is known to accelerate the binding of the pathogenic virus to their target cells and enhance inflammatory response leading to a cytokine storm [18].

In Lithuania, the first case of SARS-CoV-2 infection was officially reported on 28 February 2020, and the first case of COVID-19 infection in the Vilnius region was reported on 13 March 2020 [19]. A total of 274 783 confirmed cases and nearly 4300 deaths have been reported until 1 June 2021 [20]. It is important to know clinical characteristics and factors associated with poor outcomes of COVID-19 patients in every region and country to create the effective patient monitoring and treatment strategy and provide individually and economically appropriate healthcare. To the best of the author’s knowledge, this kind of data of Lithuanian population has not been published yet. Therefore, the aim of our study was to identify the main predictors associated with in-hospital mortality due to COVID-19 infection in Vilnius, Lithuania.

## Materials and methods

### Study design and setting

This is a retrospective observational cohort study conducted at a tertiary care university hospital Vilnius University Hospital Santaros Clinics, Vilnius, Lithuania. The objectives of our study were: to describe main clinical and initial laboratory characteristics of hospitalized COVID-19 positive patients, to obtain optimal cut-off values and predictive accuracy for in-hospital mortality due to COVID-19 infection of initial laboratory tests performed on admission and to identify the main predictors associated with in-hospital mortality due to COVID-19 infection.

### Participants

Inclusion criteria were people older than 18 years who were hospitalized to any COVID-19 unit, including standard care, high dependency, or intensive care unit, in Vilnius University Hospital Santaros Clinics with confirmed COVID-19 infection between March 2020 and May 2021. The infection was confirmed by positive SARS-CoV-2 reverse transcriptase polymerase chain reaction or rapid antigen test in nasopharyngeal sample. The antigen test was used for symptomatic patients within 5 days from the onset of COVID-19 symptoms.

### Data collection and variables

Depersonalized data were retrieved from electronic Vilnius University Hospital Santaros Clinics medical records and were provided by Informatics and development center of Vilnius University Hospital Santaros Clinics in accordance with hospital-approved procedures.

Demographic variables included gender and age. Data about comorbidities included: arterial hypertension (coded as I10; I11.0; I11.9 according to the International Statistical Classification of Diseases and Related Health Problems, Tenth Revision, Australian Modification (ICD-10-AM)), diabetes mellitus without complications (E10.9, E11.9), diabetes mellitus with complications (E10, E11 except for E10.9, E11.9), obesity (E66, E66.0, E66.2, E66.8, E66.9), chronic kidney disease (N18.1, N18.2, N18.3, N18.4, N18.5, N18.9), heart failure (I50.0, I50.1, I50.9), HIV infection (B23.8, B20), viral hepatitis C (B18.2), viral hepatitis B (B18.1), chronic obstructive pulmonary disease (COPD) (J44.0, J44.1, J44.8, J44.9), previous stroke (I69), previous myocardial infarction (I25.2), coronary artery disease (I20.0, I20.2, I20.8, I20.9), organ transplantation (Z94.0, Z94.1, Z94.4). If underlying condition was not registered by these ICD-10-AM codes in patient medical records during hospitalization due to COVID-19, the patient was considered as not having that condition.

Information about used medications (antibiotics, systemic steroids, antivirals), required invasive mechanical ventilation, and length of hospitalization was also obtained from depersonalized electronic medical records.

The results of initial laboratory tests that were performed upon admission to hospital, including complete blood count, glucose, creatinine, urea, sodium, potassium, alanine aminotransferase (ALT), aspartate aminotransferase (AST), lactate dehydrogenase (LDH), C-reactive protein (CRP), ferritin, interleukin 6 (IL-6), D-dimer, fibrinogen, troponin I, were also extracted and evaluated.

The main outcome in this study was in-hospital mortality. Therefore, the patients were distributed into two groups according to their outcome. Patients who died during the hospitalization due to COVID-19 infection were included in the ‘Lethal outcome’ group. Patients who were discharged or transferred to another hospital or nursing care facility were included into ‘Non-lethal outcome’ group.

### Statistical analysis

Continuous variables are presented as median (interquartile range (IQR)). For categorical variables, absolute and relative frequencies were calculated. Mann-Whitney U test was used to compare continuous variables, and χ^2^; test, or Fisher’s exact test was used to compare categorical variables. The predictive value of laboratory parameters was evaluated by measuring the area under the receiver operating characteristic (ROC) curve. The optimal cut-off value was obtained by calculating the Youden index (sensitivity + specificity–1). To explore the predictors associated with in-hospital mortality due to COVID-19, univariable and multivariable binary logistic regression models were created. Comorbidities statistically significantly associated with in-hospital mortality in univariable analysis, age, gender, and concentration of laboratory tests analytes with highest established predictive accuracy (as a categorical variable dichotomized according to the cut-off value) were included into multivariable analysis. Odds ratio (OR), 95% confidence interval (CI) were reported for logistic regression. Sensitivity analysis was conducted by calculating E-value for OR and lower limit of CI [21]. *p*-value <0.05 indicated statistical significance. IBM Statistical Package for the Social Sciences software version 20.0 was used for statistical analysis.

### Ethical aspects

The study was conducted in accordance with the Declaration of Helsinki. In this study, only depersonalized and pseudonymized data provided by Informatics and development center of Vilnius University Hospital Santaros Clinics in accordance with hospital-approved procedures were analyzed. For this reason, no bioethics approval is required for this study. The need for participant consent was waived because only depersonalized and strictly pseudonymized data was analyzed. Vilnius University Hospital Santaros Clinics have confirmed that data processing for this biomedical research was carried out and this publication was released under the Law on the Ethics of Biomedical Research of the Republic of Lithuania (2022-12-13; No. SR-7021).

## Results

### Demographic, clinical, and laboratory characteristics of hospitalized COVID-19 patients

**Fig 1:**
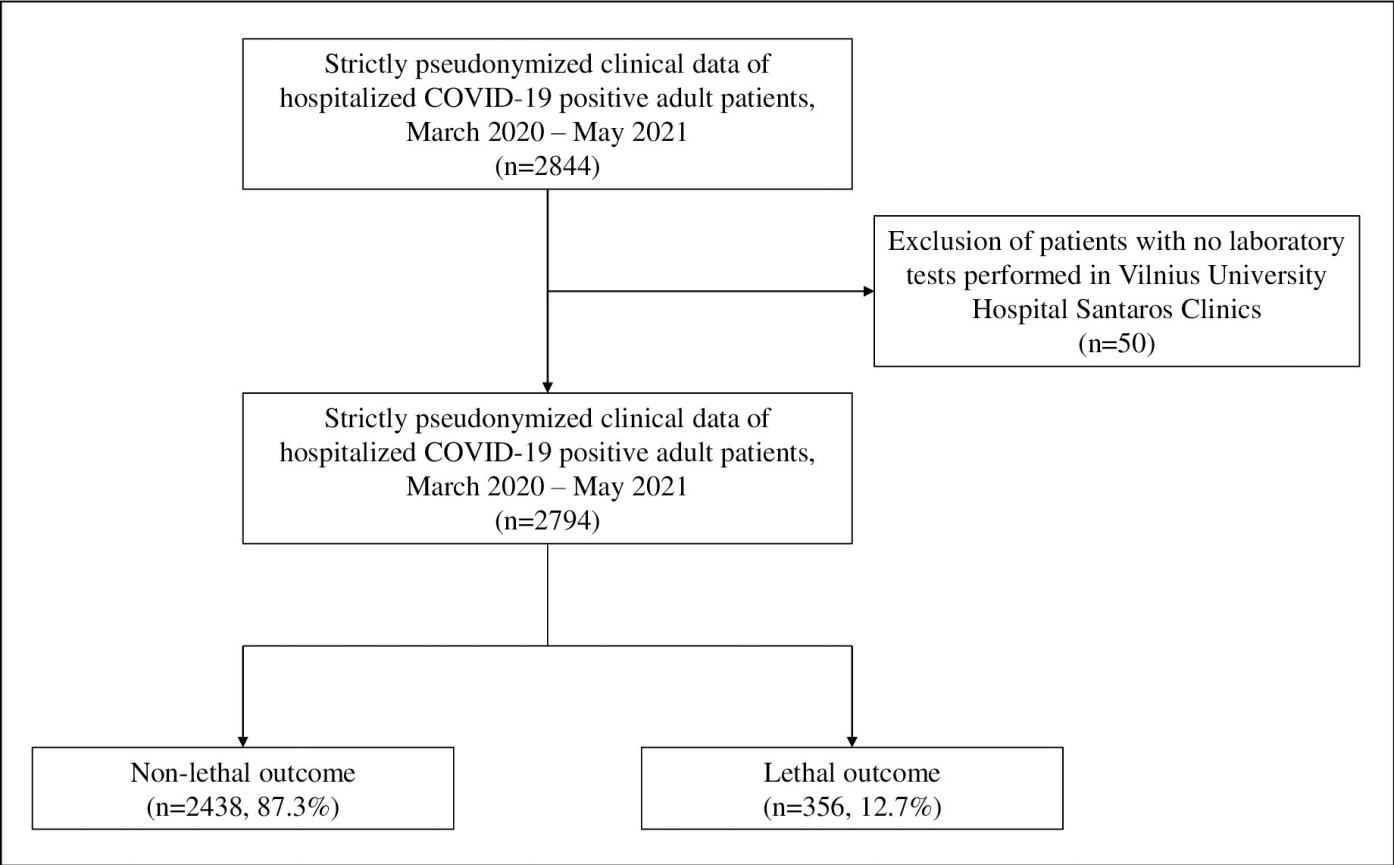
The flow chart of patient selection.

Overall, 2794 hospitalized COVID-19 positive adults were included in this study. The flow chart of patient selection is presented in Fig 1.

**Fig 2:**
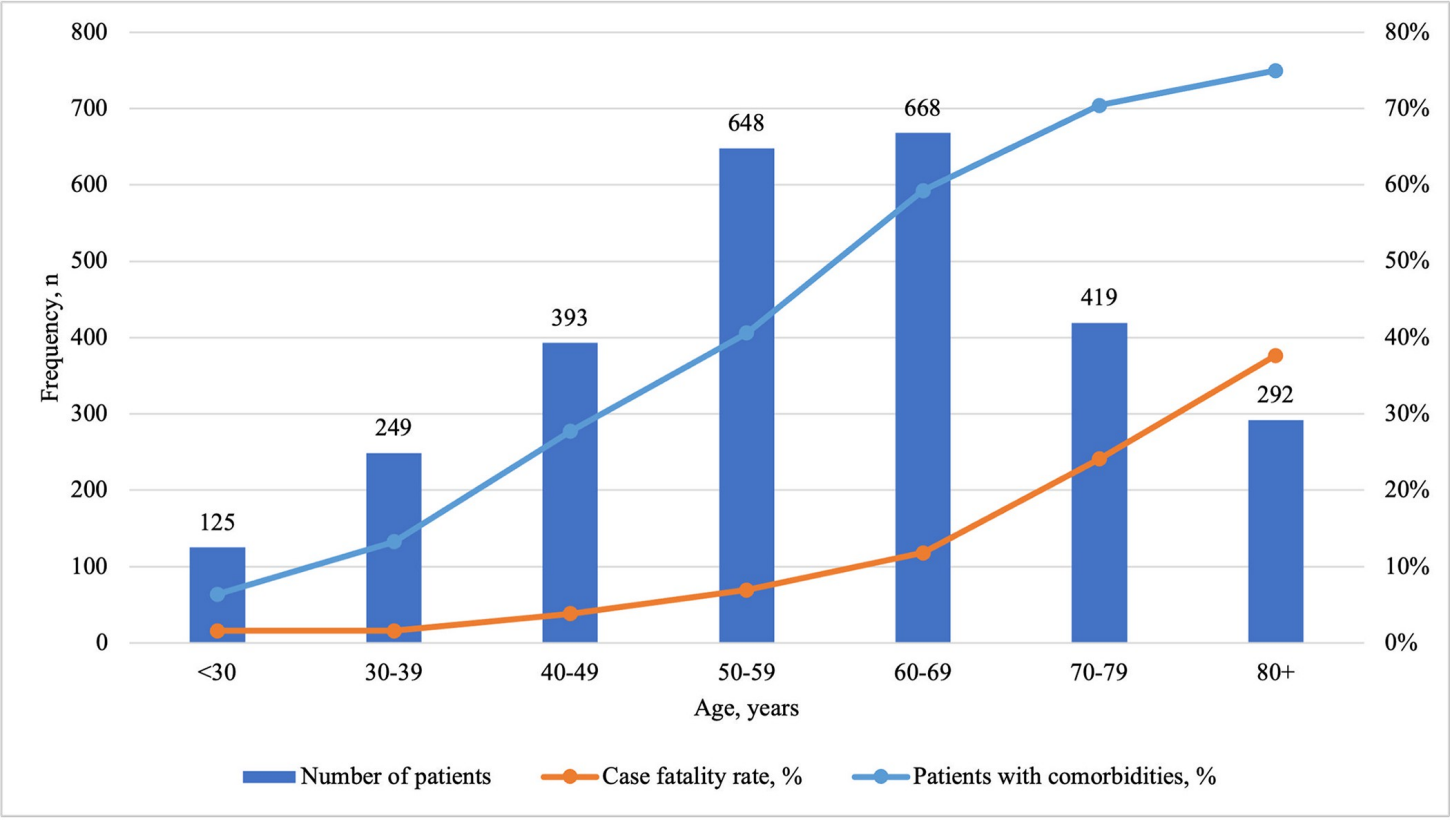
Distribution of patients and comorbidities, and case fatality rate of patients by age.

Among 2794 hospitalized patients, 54.4% were male. The median age was 59 (IQR 48–70) years, and 49.4% of patients were 60 years old and older (Fig 2).

Almost half of the patients (n = 1323/2794, 47.4%) had at least one underlying medical condition. The most frequent comorbidities were arterial hypertension (n = 1030/2794, 36.9%) and diabetes mellitus (n = 382/2794, 13.7%) (Table 1). The proportion of patients with underlying medical conditions increased with age reaching 75% of patients at age of 80 years and older (Fig 2). Prevalence of arterial hypertension, coronary artery disease, congestive heart failure, COPD, chronic kidney disease, stroke, previous transplant, HIV infection, viral hepatitis B and C did not differ between males and females. Obesity and diabetes mellitus were more prevalent among females than males (obesity 5.5% *vs* 3.9%, p = 0.043 and diabetes mellitus 16.1% *vs* 11.6%, p = 0.001, respectively).

**Table 1:** Demographic, clinical, and therapeutic characteristics of hospitalized COVID-19 patients by outcome.

Characteristic	Total (N = 2794), n (%)	Non-lethal outcome (N = 2438), n (%)	Lethal outcome (N = 356), n (%)	*p*-value
Age in years, median (IQR)	59 (48–70)	58 (47–67)	72.5 (62–81)	<0.001
Male	1520 (54.4)	1324 (54.3)	196 (55.1)	0.791
Female	1274 (45.6)	1114 (45.7)	160 (44.9)	0.791
**Comorbidities**				
Any underlying condition	1323 (47.4)	1045 (42.9)	278 (78.1)	<0.001
Arterial hypertension	1030 (36.9)	835 (34.2)	195 (54.8)	<0.001
Coronary artery disease	105 (3.8)	73 (3.0)	32 (9.0)	<0.001
Congestive heart failure	220 (7.9)	117 (4.8)	103 (28.9)	<0.001
Diabetes mellitus	382 (13.7)	297 (12.2)	85 (23.9)	<0.001
Diabetes mellitus with no complications	111 (4.0)	105 (4.3)	6 (1.7)	0.018
Diabetes mellitus with complications	272 (9.7)	193 (7.9)	79 (22.2)	<0.001
Obesity	129 (4.6)	88 (3.6)	41 (11.5)	<0.001
COPD	44 (1.6)	27 (1.1)	17 (4.8)	<0.001
Chronic kidney disease	215 (7.7)	153 (6.3)	62 (17.4)	<0.001
Previous stroke	39 (1.4)	19 (0.8)	20 (5.6)	<0.001
Transplant recipient	32 (1.1)	26 (1.1)	6 (1.7)	0.287
HIV infection	4 (0.1)	4 (0.2)	0 (0.0)	1.000
Viral hepatitis B	14 (0.5)	12 (0.5)	2 (0.6)	0.697
Viral hepatitis C	6 (0.2)	6 (0.2)	0 (0.0)	1.000
**Complications**				
Sepsis	219 (7.8)	67 (2.7)	152 (42.7)	<0.001
Acute kidney injury	296 (10.6)	112 (4.6)	184 (51.7)	<0.001
Acute myocardial infarction	47 (1.7)	40 (1.6)	7 (2.0)	0.655
Acute pulmonary embolism	63 (2.3)	45 (1.8)	18 (5.1)	<0.001
Acute stroke	39 (1.4)	30 (1.2)	9 (2.5)	0.052
**Treatment**				
Invasive mechanical ventilation	225 (8.1)	56 (2.3)	169 (47.5)	<0.001
Antibiotics	1931 (69.1)	1659 (68.0)	272 (76.4)	0.001
None	863 (30.9)	779 (32.0)	84 (23.6)	<0.001
One	1165 (41.7)	1060 (43.5)	105 (29.5)	
Two	471 (16.9)	400 (16.4)	71 (19.9)	
Three or more	295 (10.6)	199 (8.2)	96 (27.0)	
Antivirals (Remdesivir)	820 (29.3)	752 (30.8)	68 (19.1)	<0.001
Systemic steroids	1679 (60.1)	1452 (59.6)	227 (63.8)	0.130
Length of hospitalization in days, median (IQR)	11 (7–16)	11 (7–16)	12 (5.25–19)	0.360

COPD–chronic obstructive pulmonary disease; HIV–human immunodeficiency virus; IQR–interquartile range.

A total of 1931/2794 (69.1%) patients were treated with antibiotics (Table 1). The combinations of amoxicillin/clavulanic acid and piperacillin/tazobactam were the most frequently administered antibiotics given to 81.2% (1568/1931) and 32.5% (627/1931) of patients receiving antibiotics, respectively. Median duration of treatment with amoxicillin/clavulanic acid was 7 (IQR 4–10) days, and with piperacillin/tazobactam– 8 (IQR 5–11) days. Sixty percent of patients (1679/2794, 60.1%) received systemic steroids, 96.8% (1625/1679) of them received dexamethasone. Median duration of treatment with dexamethasone was 8 (IQR 5–10) days. Almost one-third of patients (820/2794, 29.3%) were treated with remdesivir for 5 (IQR 5–5) days. Remdesivir was administered intravenously as a 200 mg dose on day 1, followed by a 100 mg dose on days 2 through 5. Out of all the patients treated with remdesivir, 78% (640/820) completed full treatment regimen. Among 2794 hospitalized patients, 8.1% (225/2794) required invasive mechanical ventilation. Sepsis was diagnosed in 7.8% (219/2794) of patients, acute kidney injury occurred in 10.6% (296/2794), acute myocardial infarction–in 1.7% (47/2794), acute pulmonary embolism–in 2.3% (63/2794), and stroke affected 1.4% (39/2794) of patients during hospitalization.

The median length of hospitalization was 11 (IQR 7–16) days. Four-fifths of patients (2240/2794, 80.2%) were discharged home, 7.1% (198/2794) of patients were transferred to another hospital or nursing home, 12.7% (356/2794) of patients died. Case fatality rate increased with age from 1.6% in patients under the age of forty to 37.1% in patients at age of eighty years and older (Fig 2).

Initial laboratory results and the comparison of their medians by outcome groups are summarized in Table 2.

**Table 2:** Initial laboratory characteristics of hospitalized COVID-19 patients by outcome.

Variable	All patients		Non-lethal outcome		Lethal outcome		*p*-value
	n	Value, median (IQR)	n	Value, median (IQR)	n	Value, median (IQR)	
Hemoglobin, g/L	2794	137 (123–149)	2438	138 (125–149)	356	127 (111–145)	<0.001
WBC, x10^9^/L	2794	6.58 (4.89–9.25)	2438	6.43 (4.83–8.77)	356	8.56 (5.67–12.00)	<0.001
Neutrophils, x10^9^/L	2794	4.84 (3.33–7.30)	2438	4.67 (3.24–6.80)	356	6.96 (4.38–10.28)	<0.001
Lymphocytes, x10^9^/L	2794	1.00 (0.70–1.41)	2438	1.04 (0.75–1.48)	356	0.72 (0.50–1.10)	<0.001
NLR	2791	4.69 (2.80–8.12)	2437	4.40 (2.69–7.28)	354	8.85 (4.61–17.70)	<0.001
Platelets, x10^9^/L	2794	199.0 (155.0–259.0)	2438	200.5 (157.0–260.0)	356	192.0 (143.0–255.8)	0.010
Glucose, mmol/L	2589	6.18 (5.50–7.35)	2268	6.11 (5.45–7.11)	321	7.16 (6.03–9.39)	<0.001
Creatinine, μmol/L	2741	81.00 (66.00–104.11)	2386	80.00 (65.24–97.66)	355	110.00 (78.00–170.29)	<0.001
Urea, mmol/L	2509	5.77 (4.14–8.90)	2157	5.38 (3.99–7.66)	352	11.67 (7.10–20.68)	<0.001
Sodium, mmol/L	2743	140.00 (137.00–143.00)	2386	140.00 (137.00–143.00)	357	138.00 (134.95–142.00)	<0.001
Potassium, mmol/L	2743	4.2 (3.9–4.6)	2386	4.2 (3.9–4.5)	357	4.3 (3.9–4.8)	0.003
ALT, U/L	2647	31.04 (19.00–52.00)	2312	31.20 (19.85–51.96)	335	31.00 (17.00–55.00)	0.669
AST, U/L	2629	36.00 (25.21–57.00)	2293	35.00 (25.00–53.00)	336	51.81 (31.09–78.75)	<0.001
AST to ALT ratio	2629	1.18 (0.88–1.68)	2292	1.13 (0.85–1.57)	334	1.69 (1.19–2.49)	<0.001
LDH, U/L	2404	303 (235–411)	2113	294 (231–392)	291	417 (298–626)	<0.001
CRP, mg/L	2775	59.60 (19.90–123.00)	2419	53.30 (17.50–111.80)	356	118.40 (58.53–183.43)	<0.001
Ferritin, μg/L	2351	481.50 (236.26–1019.25)	2143	447.51 (224.00–954.00)	319	717.00 (366.60–1687.24)	<0.001
IL-6, ng/L	2351	29.50 (14.30–57.40)	2041	27.00 (13.50–52.25)	310	55.15 (26.18–122.50)	<0.001
D-dimer, μg/L	2436	522.50 (310.00–1045.00)	2112	480.00 (290.00–888.75)	324	1080.00 (560.00–2158.75)	<0.001
Fibrinogen, g/L	2146	5.42 (4.47–6.53)	1853	5.39 (4.51–6.46)	293	5.68 (4.12–6.93)	0.400
Troponin I, ng/L	2179	10.00 (5.18–27.00)	1897	9.00 (5.00–20.65)	282	41.00 (15.75–173.50)	<0.001

ALT–alanine aminotransferase; AST–aspartate aminotransferase; CRP–C-reactive protein; IL-6 –interleukin 6; IQR–interquartile range; LDH–lactate dehydrogenase; NLR–neutrophil-to-lymphocyte ratio; WBC–white blood cell count.

Reference values: hemoglobin: 128–160 g/L (for males), 117–145 g/L (for females); WBC: 4.0–9.8 x10^9^/L; neutrophils: 1.5–6.0 x10^9^/L; lymphocytes: 1.0–4.0 x10^9^/L; NLR– 1–2 [22]; platelets: 140–450 x10^9^/L; glucose: 4.2–6.1 mmol/L; creatinine: 64–104 μmol/L (for males), 49–90 μmol/L (for females); urea: 2.5–7.5 mmol/L; sodium: 134–145 mmol/L; potassium: 3.8–5.3 mmol/L; ALT: ≤40 U/l; AST: ≤40 U/L; AST to ALT ratio: <1; LDH: 125–243 U/L; CRP: <5 mg/L; ferritin: 25–350 μg/L (for men), 13–232 μg/L (for women); IL-6: 0–7 ng/L; D-dimer: <250 μg/L; fibrinogen: 2–4 g/L; troponin I: <19 ng/L.

### Clinical and laboratory characteristics associated with lethal outcome of COVID-19 patients

Compared with patients in non-lethal group, patients with lethal outcome during hospitalization were older (72.5 (62–81) years *vs* 58 (47–67) years, p<0.001), and more often had comorbidities such as arterial hypertension (54.8% *vs* 34.2%, p<0.001), coronary artery disease (9.0% *vs* 3.0%, p<0.001), congestive heart failure (28.9% *vs* 4.8%, p<0.001), diabetes mellitus (23.9% *vs* 12.2%, p<0.001), obesity (11.5% *vs* 3.6%, p<0.001), COPD (4.8% *vs* 1.1%, p<0.001), chronic kidney disease (17.4% *vs* 6.3%, p<0.001), and prior stroke (5.6% *vs* 0.8%, p<0.001) (Table 1).

On admission, patients with lethal outcome had higher levels of white blood cell count (WBC), neutrophil count, neutrophil-to-lymphocyte ratio (NLR), glucose, creatinine, urea, potassium, AST to ALT ratio, LDH, CRP, ferritin, IL-6, D-dimer, and troponin I compared to those in non-lethal group. Whereas, haemoglobin concentration, lymphocytes count, platelets, and sodium concentration were significantly lower in non-survivors. Only the medians of ALT and fibrinogen concentrations did not differ between these groups. These differences are detailed and presented in Table 2.

**Fig 3:**
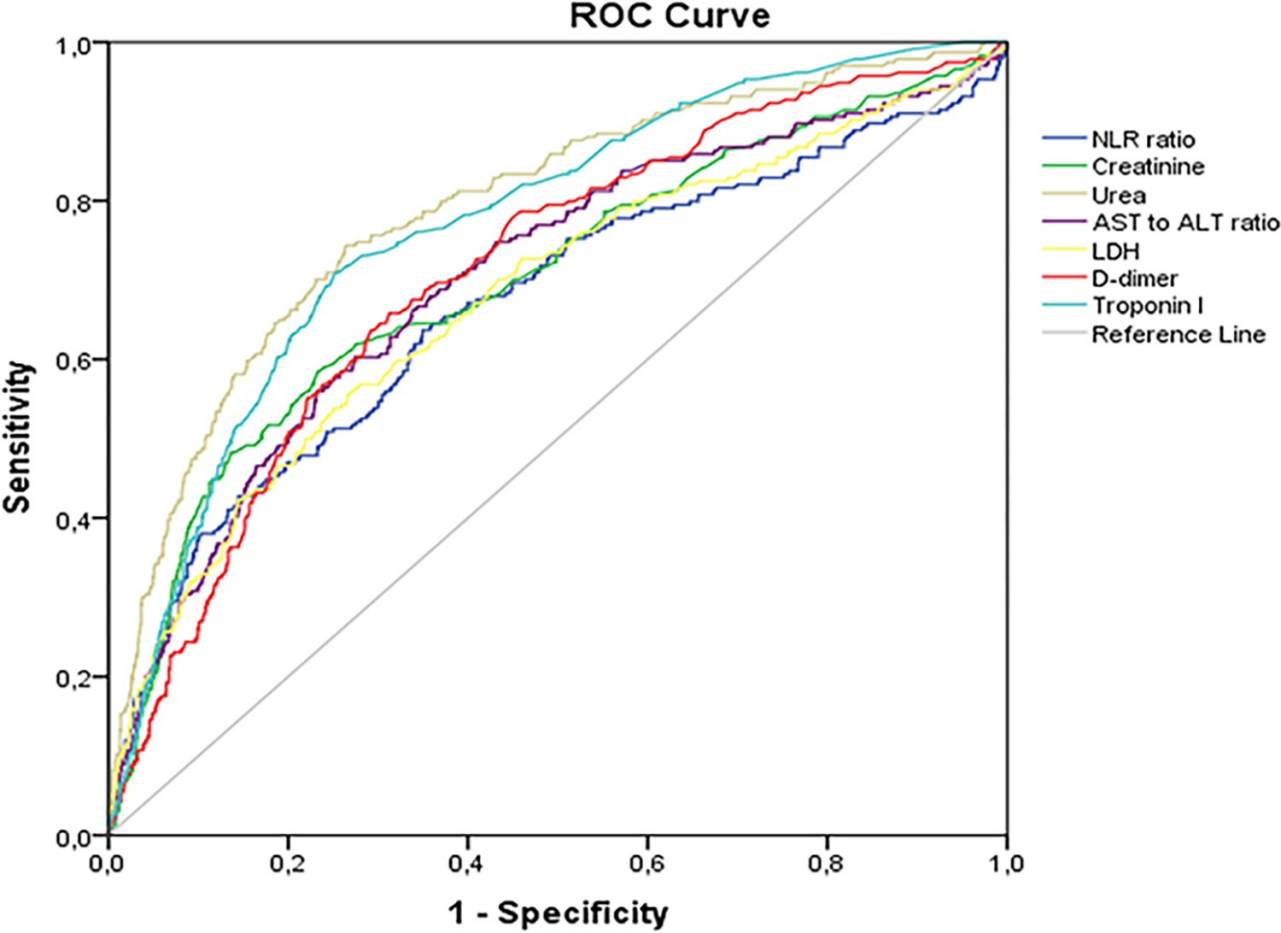
Predictive accuracy for in-hospital mortality of initial laboratory tests performed upon admission, ROC curve. ALT–alanine aminotransferase; AST–aspartate aminotransferase; LDH–lactate dehydrogenase; NLR–neutrophil-to-lymphocyte ratio.

The area under the ROC curve (AUC) were calculated to evaluate predictive accuracy of laboratory tests for lethal outcome. These results are presented in Table 3 and Fig 3. Urea possessed the highest predictive accuracy with AUC 0.80 (95% confidence interval (CI) 0.77–0.82), NLR, creatinine, AST to ALT ratio, LDH, D-dimer, and troponin I demonstrated good predictive accuracy with AUC ≥0.70, CRP, and IL-6 showed fair predictive accuracy with AUC 0.68 (95% CI 0.65–0.71) and 0.69 (95% CI 0.66–0.72), respectively. Optimal cut-off values were calculated and are shown in Table 3.

**Table 3:** Predictive accuracy for in-hospital mortality of initial laboratory tests performed upon admission.

Variable	AUC (95% CI)	*p*-value	Cut-off value	Reference value
Hemoglobin, g/L	0.40 (0.36–0.43)	<0.001	172.5	128–160 (for males) 117–145 (for females)
WBC, x10^9^/L	0.63 (0.60–0.67)	<0.001	8.09	4.0–9.8
Neutrophils, x10^9^/L	0.66 (0.62–0.69)	<0.001	6.16	1.5–6.0
Lymphocytes, x10^9^/L	0.34 (0.31–0.37)	<0.001	3.07	1.0–4.0
NLR	0.71 (0.68–0.74)	<0.001	5.71	1–2 [22]
Platelets, x10^9^/L	0.46 (0.42–0.49)	0.010	332.5	140–450
Glucose, mmol/L	0.66 (0.62–0.69)	<0.001	7.07	4.2–6.1
Creatinine, μmol/L	0.70 (0.66–0.73)	<0.001	98.97	64–104 (for males) 49–90 (for females)
Urea, mmol/L	0.80 (0.77–0.82)	<0.001	7.01	2.5–7.5
Sodium, mmol/L	0.40 (0.36–0.43)	<0.001	150.7	134–145
Potassium, mmol/L	0.55 (0.51–0.58)	0.003	4.68	3.8–5.3
ALT, U/L	0.49 (0.46–0.53)	0.669	102.5	≤40
AST, U/L	0.65 (0.62–0.68)	<0.001	55.92	≤40
AST to ALT ratio	0.70 (0.67–0.73)	<0.001	1.49	<1
LDH, U/L	0.70 (0.67–0.74)	<0.001	452.50	125–243
CRP, mg/L	0.68 (0.65–0.71)	<0.001	92.86	<5
Ferritin, μg/L	0.63 (0.59–0.66)	<0.001	483.34	25–350 (for men) 13–232 (for women)
IL-6, ng/L	0.69 (0.66–0.72)	<0.001	69.55	0–7
D-dimer, μg/L	0.72 (0.69–0.75)	<0.001	687.5	<250
Fibrinogen, g/L	0.52 (0.48–0.56)	0.400	7.51	2–4
Troponin I, ng/L	0.77 (0.74–0.80)	<0.001	18.95	<19

ALT–alanine aminotransferase; AST–aspartate aminotransferase; AUC—area under the ROC curve; CI–confidence interval; CRP–C-reactive protein; IL-6 –interleukin 6; LDH–lactate dehydrogenase; NLR–neutrophil-to-lymphocyte ratio; WBC–white blood cell count.

### Predictors for COVID-19 in-hospital mortality

Univariable analysis revealed that age, arterial hypertension, coronary artery disease, congestive heart failure, diabetes mellitus, obesity, COPD, chronic kidney disease, and previous stroke were associated with in-hospital mortality in patients with COVID-19 (Table 4).

**Table 4:** Predictors associated with in-hospital mortality of hospitalized patients with COVID-19.

	Univariable regression		Multivariable regression			
Characteristic	OR (95% CI)	*p*-value	OR (95% CI)	*p*-value	E-value for OR; for CI lower limit
Age in years	1.07 (1.06–1.08)	<0.001	1.04 (1.02–1.06)	<0.001	1.24; 1.16
Male	1.03 (0.82–1.29)	0.791	0.84 (0.58–1.20)	0.329	-
**Comorbidities**					
Arterial hypertension	2.33 (1.86–2.91)	<0.001	0.79 (0.55–1.15)	0.224	-
Coronary artery disease	3.20 (2.08–4.93)	<0.001	1.19 (0.62–2.26)	0.601	-
Congestive heart failure	8.08 (6.01–10.85)	<0.001	3.06 (1.96–4.77)	<0.001	5.57; 3.33
Diabetes mellitus	2.26 (1.72–2.97)	<0.001	0.92 (0.60–1.43)	0.717	-
Obesity	3.48 (2.36–5.13)	<0.001	3.90 (2.12–7.16)	<0.001	7.26; 3.66
COPD	4.48 (2.42–8.30)	<0.001	2.92 (1.12–7.60)	0.029	5.29; 1.49
Chronic kidney disease	3.15 (2.29–4.33)	<0.001	1.00 (0.59–1.68)	0.997	-
Previous stroke	7.58 (4.00–14.35)	<0.001	5.80 (2.07–16.21)	<0.001	11.08; 3.56
Transplant recipient	1.59 (0.65–3.89)	0.309	-	-	-
Viral hepatitis B	1.15 (0.26–5.18)	0.851	-	-	-
**Laboratory analytes**					
NLR > 5.71	4.10 (3.22–5.22)	<0.001	1.09 (0.75–1.60)	0.647	-
Creatinine > 98.97, μmol/L	4.58 (3.64–5.78)	<0.001	1.23 (0.79–1.90)	0.361	-
Urea > 7.01, mmol/L	7.72 (5.94–10.03)	<0.001	2.32 (1.47–3.67)	<0.001	4.07; 2.30
AST to ALT ratio > 1.49	3.92 (3.09–4.97)	<0.001	1.54 (1.08–2.21)	0.018	2.54; 1.37
LDH > 452.5, U/L	4.82 (3.72–6.25)	<0.001	2.60 (1.74–3.88)	<0.001	4.64; 2.87
CRP > 92.68, mg/L	3.60 (2.86–4.53)	<0.001	1.58 (1.06–2.35)	0.024	2.54; 1.31
IL-6 > 69.55, ng/L	4.42 (3.43–5.70)	<0.001	1.62 (1.10–2.40)	0.015	2.62; 1.43
D-dimer > 687.5, μg/L	4.67 (3.62–6.02)	<0.001	1.14 (0.78–1.66)	0.492	-
Troponin I > 18.95, ng/L	7.11 (5.38–9.41)	<0.001	2.04 (1.38–3.02)	<0.001	3.5; 2.1

ALT–alanine aminotransferase; AST–aspartate aminotransferase; CI–confidence interval; COPD–chronic obstructive pulmonary disease; CRP–C-reactive protein; IL-6 –interleukin 6; LDH–lactate dehydrogenase; NLR–neutrophil-to-lymphocyte ratio; OR–odds ratio.

Reference values: NLR– 1–2 [22]; creatinine: 64–104 μmol/L (for males), 49–90 μmol/L (for females); urea: 2.5–7.5 mmol/L; AST to ALT ratio: <1; LDH: 125–243 U/L; CRP: <5 mg/L; IL-6: 0–7 ng/L; D-dimer: <250 μg/L; troponin I: <19 ng/L.

**Fig 4:**
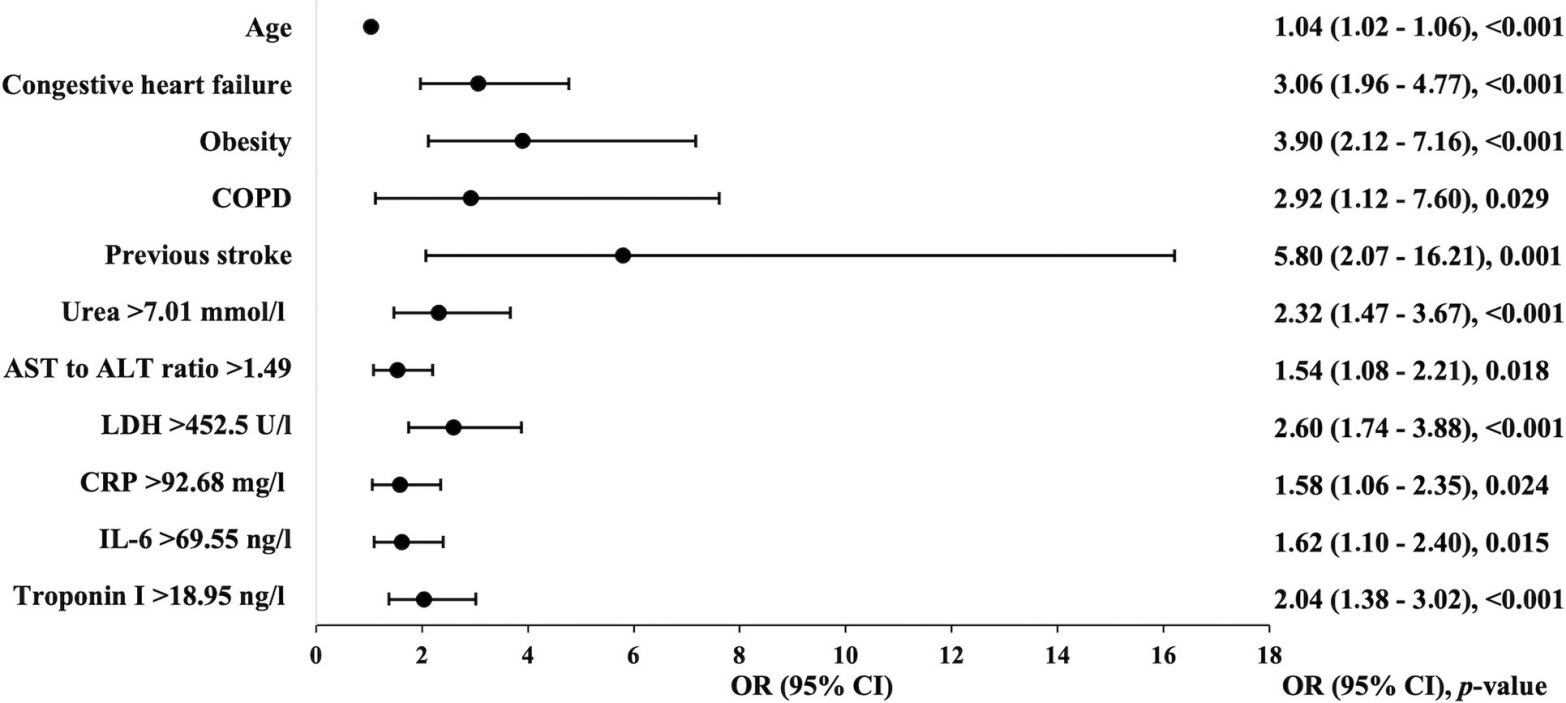
Significant predictors associated with in-hospital mortality in patients with COVID-19, multivariable logistic regression model. ALT–alanine aminotransferase; AST–aspartate aminotransferase; CI–confidence interval; COPD–chronic obstructive pulmonary disease; CRP–C-reactive protein; IL-6 –interleukin 6; LDH–lactate dehydrogenase; OR–odds ratio.

Multivariable analysis confirmed that age (OR 1.04, 95% CI 1.02–1.06), congestive heart failure (OR 3.06, 95% CI 1.96–4.77), obesity (OR 3.90, 95% CI 2.12–7.16), COPD (OR 2.92, 95% CI 1.12–7.60), previous stroke (OR 5.80, 95% CI 2.07–16.21) and the following laboratory parameters: urea >7.01 mmol/l (OR 2.32, 95% CI 1.47–3.67), AST to ALT ratio >1.49 (OR 1.54, 95% CI 1.08–2.21), LDH >452.5 U/l (OR 2.60, 95% CI 1.74–3.88), CRP > 92.68 mg/l (OR 1.58, 95% CI 1.06–2.35), IL-6 > 69.55 ng/l (OR 1.62, 95% CI 1.10–2.40), and troponin I >18.95 ng/l (OR 2.04, 95% CI 1.38–3.02) are independent predictors for in-hospital mortality of patients with COVID-19 (Table 4, Fig 4). The E-value was calculated and attested the robustness of predictors and in-hospital mortality association regarding bias caused by potential unmeasured confounders (Table 4).

**Fig 5:**
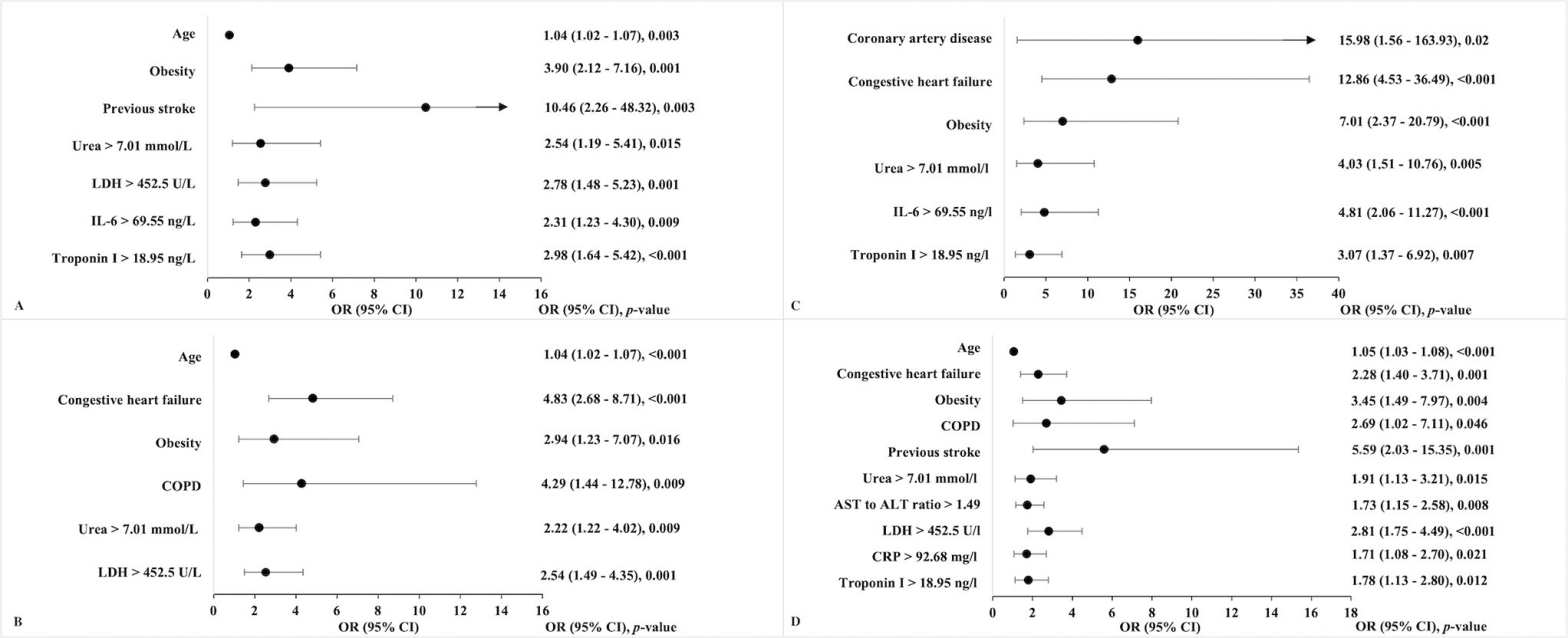
Significant predictors associated with in-hospital mortality in patients with COVID-19, stratified by age and sex, multivariable logistic regression models. (A) Predictors associated with in-hospital mortality in female COVID-19 patients. (B) Predictors associated with in-hospital mortality in male COVID-19 patients. (C) Predictors associated with in-hospital mortality in COVID-19 patients aged 60 years and younger. (D) Predictors associated with in-hospital mortality in COVID-19 patients aged over 60 years. ALT–alanine aminotransferase; AST–aspartate aminotransferase; CI–confidence interval; COPD–chronic obstructive pulmonary disease; CRP–C-reactive protein; IL-6 –interleukin 6; LDH–lactate dehydrogenase; OR–odds ratio.

Univariable and multivariable analyses were repeated in subgroups stratified by sex and by age (Fig 5). In multivariable analysis age, obesity, urea >7.01 mmol/L, and LDH >452.5 U/L were associated with higher risk for in-hospital mortality in patients of both genders, while congestive heart failure (OR 4.83, CI 95% 2.68–8.71) and COPD (OR 4.29, CI 95% 1.44–12.78) were identified as predictors only in male COVID-19 patients, and previous stroke (OR 10.46, CI 95% 2.26–48.32), IL-6 > 69.55 ng/L (OR 2.31, CI 95% 1.23–4.30) and troponin I >18.95 ng/L (OR 2.98, CI 95% 1.64–5.42)–only in female patients.

Multivariable analysis of subgroups by age revealed that congestive heart failure, obesity, urea >7.01 mmol/L, and troponin I >18.95 ng/L were associated with in-hospital mortality of COVID-19 patients in both groups–patients aged 60 years or younger and patients over 60 years old. Coronary artery disease (OR 15.98, 95% CI 1.56–163.93), IL-6 > 69.55 ng/L (OR 4.81, 95% CI 2.06–11.27) were identified as predictors for in-hospital mortality in COVID-19 patients aged 60 years and below, while age (OR 1.05, 95% CI 1.03–1.08), COPD (OR 2.69, 95% CI 1.02–7.11), previous stroke (OR 5.59, 95% CI 2.03–15.35), AST to ALT ratio >1.49 (OR 1.73, 95% CI 1.15–2.58), LDH >452.5 U/L (OR 2.81, 95% CI 1.75–4.49), and CRP > 92.68 mg/L (OR 1.70, 95% CI 1.08–2.70) were also associated with in-hospital mortality in patients older than 60 years old.

## Discussion

In this retrospective study, we report the patient characteristics and predictors associated with in-hospital mortality among hospitalized COVID-19 patients in one of the main tertiary care hospitals in the country. This is one of the few studies on this topic conducted in Eastern Europe and particularly in the Baltic region. This study contributes to the knowledge of the natural course of SARS-CoV-2 infection and how gender, age, underlying conditions, and laboratory characteristics are associated with the outcomes of the disease. We identified age, congestive heart failure, obesity, COPD, prior stroke, and increased concentration of urea (> 7.01 mmol/L), LDH (> 452.5 U/L), CRP (> 92.68 mg/L), IL-6 (> 69.55, ng/L), troponin I (> 18.95 ng/L), ALT to AST ratio (> 1.49) to be predictors for in-hospital mortality of COVID-19 patients. These predictors slightly differed between patient groups by sex and age.

We found the in-hospital mortality to be 12.7%, which was close to Fakih et al. research (13%) conducted in the US [23] and lower than published by Gujski et al. in Poland (18.4%) [24]. Other studies done in the Netherlands, United Kingdom, Germany reported in-hospital mortality to be 24.6%, 26%, 17.9%, respectively [13, 25, 26]. It also should be noted that in-hospital mortality differed during separate COVID-19 waves and in different populations [27–29]. Gray et al. revealed that in-hospital mortality rates were lower in the second wave compared to the first for all socioeconomic and demographic groups [27]. In addition, in-hospital mortality was 15.1%, 12.6%, 12.8% during the first, the second and the third waves of COVID-19 in Spain, and 9.5%, 10.2%, 5.4% in Switzerland, respectively [28, 29]. On the contrary, Gujski et al. noted a higher fatality rate during the second COVID-19 wave in Poland (8.3% *vs* 21%) [24], which could be linked to an increased number of hospitalized patients during the second wave. Unfortunately, we could not distinguish the separate COVID-19 waves to compare the mortality rates and patient characteristics, since we did not identify different SARS-CoV-2 strains and we can only hypothesize that our study period included B.1.1.280, B.1.177.60, alpha (B.1.1.7), and B.1.620 variants.

Lower mortality could be a possible result of introduction of interventions such as remdesivir, dexamethasone, high-use of thromboprophylaxis, as well as ventilation management, and the start of COVID-19 vaccination program [30, 31]. Despite declining mortality rates, advanced age, male sex, and pre-existing comorbidity remained a key mortality risk factors among COVID-19 patients [27–29].

Consistent with other studies [25, 32–35], we found that advanced age is an independent predictor for in-hospital mortality associated with 4% increase in odds ratio per each year. This tendency could be explained by the fact that older people have more comorbidities which themselves are risk factors for in-hospital mortality of COVID-19 patients [18, 36]. This also could be due to immunosenescence, characterized by impaired age-dependent defects in T-cell and B-cell function, which weaken immune responses to most viruses, including SARS-CoV-2 [37]. Although, numerous studies have reported that males are at higher risk of severe COVID-19 infection, as well as of death, especially over 65 years [33, 38, 39], we found that 55.1% of fatal cases were male but males did not appear to have a significantly higher in-hospital mortality risk.

Docherty et al. established that up to 77.5% of hospitalized COVID-19 patients had at least one comorbidity [13]. Additionally, Thakur et al. disclosed that arterial hypertension, obesity, and diabetes mellitus were the most prevalent comorbidities [40], accounting for up to 51.3%, 35.0%, and 49.8% of cases, respectively [23, 41]. In our study, 47.4% of patients had at least one underlying condition with the most prevalent being arterial hypertension (36.9%) and diabetes mellitus a second (13.7%). Different from other researches [23, 32, 42], the prevalence of obesity in our study was considerably lower (4.6%). This difference could be related to the fact that we analyzed depersonalized database and obesity might be not encoded in patients’ medical records during this hospitalization. Patients with comorbidities are not only more vulnerable to COVID-19 infection, but underlying conditions act as triggers for increased risk of fatality [18, 36]. We observed that comorbidity was significantly associated with fatal outcome. Moreover, multivariable analysis identified congestive heart failure, obesity, COPD, and previous stroke as predictors for in-hospital mortality. While analysing the results stratified by sex, we discovered that cardiovascular and chronic lung diseases were associated with higher in-hospital mortality in men. These results are in contrast to the findings of Fernández-Martínez et al. [43] and could be linked to gender-related lifestyle differences.

We found that the presence of congestive heart failure 3.06 times increased the risk of in-hospital mortality in general population and in both groups stratified by age. Increased mortality in congestive heart failure patients is associated with dysregulation of intracellular calcium handling system, therefore COVID-19 infection causes hypoxia induced excessive intracellular calcium that culminate in apoptosis of cardiac myocyte [44]. A global meta-analysis by Popkin et al. demonstrated that obesity increased COVID-19 mortality by 1.48 times [45], whereas we found that in-hospital mortality was 3.90-fold higher in obese patients. Obesity also remained significant in subgroup analysis by sex and by age. Not only does obesity lead to an increased expression of inflammatory molecules, but also reduces thoracic wall compliance and functional residual capacity, promoting the development of acute respiratory distress syndrome related lung damage in patients with COVID-19 [46–48]. Our study showed that COPD increased in-hospital mortality in COVID-19 patients by 2.92-fold and in male patients population–by 4.29-fold. Pranata et al. also found that COVID-19 patients with COPD had a 4.36 times higher rate of death [49]. This is due to an increased expression of ACE2 in COPD epithelial cells, reduced antiviral responses (especially interferons), and the potential for secondary bacterial infection [50, 51]. We also found that previous stroke increased in-hospital mortality by 5.80 times, however the results must be taken with caution given the wide confidence interval. The relationship between death and previous stroke might be due to lasting disabilities in this group [52]. Tehrani et al. suggested that patients with a history of stroke carry a predisposition that, under the impact of COVID-19 induced coagulopathy, may lead to vascular events causing fatal outcome [52].

In this study, there were significant differences in the initial laboratory parameters between patients with lethal and non-lethal outcomes, indicating higher levels of inflammation, cell-turnover and metabolic dysregulation in the first group [53]. Many studies have investigated the role of various biomarkers in determining prognosis for patients with COVID-19 and evidenced that routine laboratory parameters have important clinical application value in predicting the course of COVID-19 [54–56]. Since the beginning of the pandemic, CPR, ferritin, LDH and D-dimer have been widely used for risk stratification, however there is marked diversity in cut-off values [39, 57, 58].

Our study results demonstrated that NLR, urea, creatinine, AST to ALT ratio, LDH, D-dimer, CRP, IL-6 and troponin I demonstrated good predictive accuracy for in-hospital mortality. Multivariable analysis confirmed elevated urea, AST to ALT ratio, LDH, CRP, IL-6, and troponin I together with age and presence of comorbidities described above to be predictors for in-hospital mortality.

The results of this study indicated that increased concentration of urea (above 7.01 mmol/L) was significantly associated with in-hospital mortality increasing these odds 2.32 times and it also remained a significant predictor in further subgroup analysis by sex and age. A study conducted in China on 12,413 patients had evidenced association of elevated blood urea nitrogen (BUN) and elevated serum creatinine with all-cause in-hospital mortality risk, respectively BUN adjusted hazard ratio (aHR) was 6.27, and serum creatinine aHR was 2.65 [59]. Moreover, Liu YM et al. reported elevated BUN level to show a more significant association with adverse outcomes than serum creatine suggesting that the elevation in BUN level not only indicates a kidney dysfunction, but it can also reflect inflammatory status, catabolism, and renal hypoperfusion from hypovolemia, sepsis, or reduced cardiac output while serum creatinine mainly represents a status of kidney injury and metabolic disturbance [59].

Furthermore, our research demonstrated that AST to ALT ratio has better predictive accuracy for in-hospital mortality than AST or ALT separately, and patients with AST to ALT ratio >1.49 had 54% higher odds for lethal outcome. Consistent results were reported in other studies [60–62].

This study revealed elevated LDH, CRP and IL-6 to be also associated with an increased risk of in-hospital mortality. Patients with LDH >452.5 U/L on admission had 2.6 times higher odds for in-hospital mortality. In a pooled analysis of nine published studies conducted by Henry et al. which included 1532 patients with COVID-19, elevated LDH levels were associated with 6-fold increased odds of developing severe disease and 16-fold increased odds of mortality that is much higher compared to our results [63]. Our findings indicated that CRP >92.68 mg/L and IL-6 >69.55 ng/L were the optimal cut-off values discriminating patients with lethal and non-lethal outcome. Both CRP and IL-6 remained predictors for in-hospital mortality in multivariable analysis that increased this risk for 58% and 62%, respectively. In multiple studies exploring routine laboratory parameters in COVID-19 patients, increased CRP levels and IL-6 were also reported in severe patients when compared to non-severe patients [64–67].

Moreover, we identified that patients with troponin I concentration above 18.95 ng/L had 2.04 times higher risk for in-hospital lethal outcome. Other studies also found out increased level of troponin to be associated with this outcome [68, 69]. The metanalysis of 12,262 COVID-19 patients indicated that increased troponin concentration was detected in 31% of patients and they had 4.75 times higher odds for mortality [70].

The combination of patient medical history and routine laboratory tests are simple, and cost-effective indicators that can be used to identify COVID-19 patients at a greater risk of death. The presence of these predictors might assist in choosing of medical care and treatment for COVID-19 patients individually.

### Bias/Limitations

Data for this study was retrieved from strictly pseudonymized and depersonalized electronic medical records based on encoded medical information. For this reason, there is a probability that some information about patients could be not mentioned or missed in their medical records or were encoded in any other code that was not extracted from electronic database. Furthermore, we could not extract the data about addictions, vaccination status, COVID-19 pandemic wave etc. and include these variables into analysis. It indicates the need of further prospective research of COVID-19 positive patients’ cohorts to get more accurate and extensive results.

As in this study we included all hospitalized COVID-19 positive adults despite their comorbidities, some laboratory test results (e.g., urea, troponin I, AST to ALT ratio) could be increased not only because of the COVID-19 infection itself, but also due to comorbidities. Therefore, the identified predictors of in-hospital mortality could be determined not only by factors caused by COVID-19 disease, but the consequences of the general condition. In the context of COVID-19 disease, laboratory markers (e.g., urea, creatinine, AST, ALT, CRB, LDH, troponin I) serve as indicators of disease severity and prognosis, while it should be recognized that these markers can be influenced by other underlying conditions, especially in hospitalized patients with COVID-19.

## Conclusions

The predictors for in-hospital mortality of COVID-19 patients were identified to be age, congestive heart failure, obesity, COPD, prior stroke, and increased concentration of urea, LDH, CRP, IL-6, troponin I, ALT to AST ratio. This study evidenced that the history of comorbidities and routine laboratory tests can be beneficial identifying COVID-19 patients at increased risk of death and choosing the best medical care strategy.

## Supporting information

S1 Data (XLS)
